# The Incidence of Inflammatory Bowel Disease in Northern China: A Prospective Population-Based Study

**DOI:** 10.1371/journal.pone.0101296

**Published:** 2014-07-16

**Authors:** Hong Yang, Yumei Li, Wei Wu, Qingwen Sun, Yunzhong Zhang, Wei Zhao, Hongbo Lv, Qing Xia, Pinjin Hu, Haihua Li, Jiaming Qian

**Affiliations:** 1 Department of Gastroenterology, Peking Union Medical College Hospital, Beijing, China; 2 Department of Gastroenterology, Daqing Longnan Hospital, Daqing, China; 3 Department of Gastroenterology, Daqing Oilfield General Hospital, Daqing, China; 4 Department of Gastroenterology, Daqing People Hospital, Daqing, China; 5 Department of Gastroenterology, Daqing Fourth Hospital, Daqing, China; 6 Department of Gastroenterology, The First Affiliated Hospital of Sun Yat-sen University, Guangzhou, China; MOE Key Laboratory of Environment and Health, School of Public Health, Tongji Medical College, Huazhong University of Science and Technology, China

## Abstract

**Aims & Backgrounds:**

Although inflammatory bowel diseases (IBD) are emerging and increasing in China, epidemiologic data are rarely available. This study was to investigate the epidemiological and clinical characteristics of IBD in Northern China.

**Methods:**

This is a prospective, population-based study of incidence of IBD in Daqing,Heilongjiang province of Northern China from March 1, 2012 to February 28, 2013. All incident patients with IBD were clinically identified by IBD specialist group from five main General Hospitals covering the healthcare service for 1,343,364 residents in the urban areas of Daqing. IBD cases included in this study were followed-up for three months for diagnosis confirmation.

**Results:**

A total of 27 new IBD cases including 25 cases of ulcerative colitis (UC) and 2 cases of Crohn's disease (CD) were identified. The population at risk was 1,343,364 person years. Age-adjusted incidence for total IBD, CD and UC were 1.77, 0.13, and 1.64 per 100,000population, respectively. A male predominance was found in CD patients (male to female ratio was 2∶0). In contrast, no obvious gender predominance was found in UC patients (male to female ratio was 1∶1.1). CD patients were diagnosed at an average age of 39.5 years. The main disease phenotypes of UC were distal colitis with a 24% of proctitis and 56% of left-sided colitis. The mean diagnostic age of UC patients was 48.9 years.

**Conclusions:**

This is the first report on the incidence of IBD in the Northern Chinese population. A lower incidence of IBD, similar male predominance for CD, similar disease phenotype of UC, and lower disease activity was observed in Daqing compared to that in Southern China.

## Introduction

Inflammatory bowel diseases (IBDs), including ulcerative colitis (UC) and Crohn's disease (CD), frequently progress with long-term disability, morbidity and a variety of complications, which lead to low quality of life in patients [1]. Recent studies demonstrated that the incidence of IBD is increasing worldwide. The highest incidence rate of IBD was reported in North America and Europe. The annual incidence of CD in North America is 20.2 per 100,000 persons and the annual incidence of UC in Europe is 24.3 per 100,000 persons. However, considerable variations in IBD incidence have been reported around the world, which was proposed to be associated with different geographic regions, environmental factors and dietary factors [2]–[5]. Few epidemiologic studies have been conducted in developing countries, such as China, South Korea, India, Lebanon, Iran, Thailand, the French West Indies, and North Africa [6]. A population-based epidemiologic study of IBD in a country with a large demographic base, such as China with a population of 1.3 billion, is invaluable for understanding the burden of disease and formulating insurance policy for health care.

The incidence of IBD has been reported from two areas of Southern China recently [7]–[8]. However, there are a number of differences in climate, environment, lifestyle, diet and living conditions between Northern and Southern China. Based on a previous report from Europe, there are geographic variations in IBD risk [9]. Therefore, it is important to analyze the epidemiological characteristics of IBD in the Northern Chinese population and identify the clinical characteristics.

In this study, a prospective, population-based study was conducted to investigate the incidence and clinical characteristics of IBD in an area of Northern China and to give an overview of IBD between different geographic regions in China.

## Materials and Methods

### Study population

This study was approved by the Institutional Review Board of Peking Union Medical College Hospital. Written informed consent was obtained from all participants. Daqing, a prefectural-level city, is located in Heilongjiang province, Northern China with a total area of 22,161 square kilometers. This area is characterized by a relative low floating population. Therefore, it is easy to monitor and follow-up. Demographic data of the study population was extracted from the 2010 Population census published by the National Bureau of Statistics of China. Based on the data within the census, the total population of Daqing is 1,585,162 and 15.25% (241,798) of the population lived in rural areas, including the countryside and small towns. Thus, a population of 1,343,364 living in the urban areas was selected as the study population for this study. The sex and age proportional composition was calculated (female for 66 0,009, and male for 683,355).

1,343,364 residents in the urban areas of Daqing are served by five general hospitals and nine community hospitals. The specialists in the gastroenterology and qualified endoscopic equipments are essential in the five general hospitals, whereas they are not in community hospitals. The community hospitals usually refer symptomatic patients to a general hospital for the disease identification and confirmation. In the five general hospitals, specialists can be found in the gastroenterology units and endoscopic unit. All residents in Daqing city are covered by social health insurance. Each resident has a medical insurance number and detailed medical record.

### Case selection procedure

**Figure 1:**
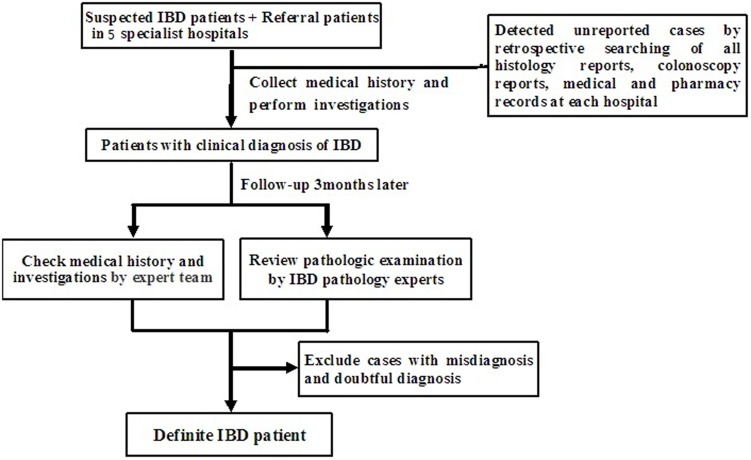
The flow chart of case capture. The flow chart described the procedure of case capture. Firstly, any patients with suspected or diagnosed IBD were checked medical history and investigations and then were follow-up for 3 months. Then expert team including IBD specialists and pathology experts determined the diagnosis. Finally, a retrospective search was performed to avoid missing cases.

The study protocol was mostly performed in adherence to published protocols in the studies of IBD in Guangzhou and Wuhan in Southern China [7]–[8] (Figure 1). Firstly, any patients with suspected or diagnosed IBD by any General Hospitals or community hospitals in Daqing were referred to Daqing Longnan Hospital for thorough Diagnosis. The demographic, clinical, endoscopic, and histologic data were collected by one of the gastroenterologists and checked by a group of gastroenterological specialists. Each participant completed the questionnaires including clinical characteristics, past history, family history, life style, and quality of life under the guidance of the IBD specialist group in Daqing Longnan Hospital.

Secondly, all the patients with suspected IBD were followed-up for 3 months. By the end of follow-up, two IBD experts determined the diagnosis for patients with suspected IBD. One pathologist reviewed pathologic results. Patients without definitive diagnosis of IBD were excluded.

Finally, to avoid missing cases, a retrospective search of local electronic medical records for endoscopic reports, medical charts, and histology reports at each hospital in Daqing was performed to find unreported incidence cases. A search using keywords such as “inflammatory bowel disease”, “Crohn's disease”, “ulcerative colitis”, “chronic colitis” was conducted and matching patient records were identified and examined. Established registry, report and management systems in each hospital of Daqing provided support for periodic retrieval and tracking of IBD cases.

### Case Definition and clinical evaluation

The diagnosis of IBD was determined according to the Lennard-Jones criteria [11]. The clinical diagnosis was confirmed on the basis of clinical symptoms, physical examination, colonoscopy, imaging (bariums studies, CT enterography), and histology. Enteric infections, intestinal tuberculosis, ischemia, non-steroidal anti-inflammatory drug induced ulceration, and radiation colitis were excluded.

Clinical classification was defined according to the Montreal Classification of IBD from [10]. Crohn's disease can be described using A, L and B classifications. A represents age at diagnosis, L represents the location of disease (A1:below 16 y; A2 between 17 and 40 y; A3 above 40 y L1: ileal;L2 colonic;L3 ileocolonic; L4 isolated upper disease). B represents disease behavior (B1:non-stricturing,non-penetrating;B2 stricturing; B3 penetrating;P perianal disease modifier). According to Montreal Classification of ulcerative colitis, the extent of UC was defined as proctitis (E1, lesions limited to the rectum), left-sided colitis (E2, lesions below the splenic flexure), and extensive/pancolitis (E3, lesions exceeded the splenic flexure).

The activity of IBD was classified according to Harvey-Bradshaw activity index for CD and SCCAI index for UC as mild, moderate and severe. The activity according to Harvey-Bradshaw was defined as remission (below 4 scores), moderate (5–8 scores), and severe (above 9 scores). The activity according to SCCAI index was defined as mild (3–5 scores), moderate (6–11 scores), and severe (above 12 scores).

### Statistical analysis

The annual crude incidence rates were calculated for IBD, UC, and CD for 2012–2013, respectively, which were calculated by the number of incident patients diagnosed divided by the total population at risk. The Age-standardized incidence rates were adjusted by the national demographic structure from 6^th^ nationwide census in 2010 (http://en.wikipedia.org/wiki/Daqing). Rates were reported with 95% confidence intervals (CI), assuming a Poisson distribution.

## Results

### 1. Incidence and age-standardized incidence for new IBD cases

27 new IBD cases including 2 CD and 25UC were diagnosed (Table 1). The median age of diagnosis was 48.2±12.3years (range, 27–79 years) for all patients. Among patients, 14 were male and 13 were female, and the ratio of males to females was 1.1∶1. Fifteen patients complete high school education or below and 12 patients completed a bachelor or higher degree. The median number of months from onset of symptoms to diagnosis of IBD was 11.0 months (interquartile range, 1–120 months).

**Table 1:** Demographic characteristics of new cases with IBD in North of China.

	IBD	UC	CD
Patients(n)	27	25	2
Gender,n(%)			
Females	13(48.1)	13(52.0)	0(0.0)
Males	14(51.9)	12(48.0)	2(100.0)
Median age at diagnosis,yr	48.2	48.9	39.5
Smoking history,n(%)			
Current	5(18.6)	4(16.0)	1(50.0)
Ex	6(22.2)	5(20.0)	1(50.0)
Never	16(59.2)	16(64.0)	0(0.0)
Education,n(%)			
Primary or below	1(3.7)	1(4.0)	0(0.0)
Secondary and apprentice	14(51.9)	13(52.0	1(50.0)
Tetiary(university or college)	12(44.4)	11(44.0)	1(500.)
Appendectomy	0(0.0)	0(0.0)	0(0.0)
Family history	0(0.0)	0(0.0)	0(0.0)
NSAID	3(11.1	2(8.0	1(50.0)
Tuberculosis history	1(3.7)	1(4.0)	0(0.0)

The 2 patients with CD were diagnosed at38 and 41 years old. Both of them are male. The months from onset of symptoms to diagnosis was 2 and 120 months, respectively. Neither of them had appendectomy, a family history of IBD, or a history of tuberculosis. One patient had taken non-steroidal anti-inflmmatory drug (NSAIDs) before.

25 patients with UC were diagnosed at diagnostic age of 48.88±12.5 years. The average diagnostic age was 48.5±15.7 years for males, and 49.23±9.2 years for females. The ratio of males to females was 12∶13. The median number of months from onset of symptoms to diagnosis of IBD was 7.04 (interquartile range, 1–48 months). None of the patients had appendectomy or a family history of UC. One patient had a history of tuberculosis. Two patients had taken NSAIDs before.

The crude annual incidence of total IBD, CD, and UC was 2.01, 0.15, and 1.86 per 100,000 population, respectively. The crude annual incidence of IBD, CD, and UC for males was 2.12, 0.30, and 1.82 per 100,000 population, respectively. Crude annual incidence for females was 1.90, 0.00, and 1.90 per 100,000 population, respectively (Table 2). Age-adjusted incidence for total IBD, CD, and UC was 1.77, 0.13, and 1.64 per 100,000 population, respectively. The age-adjusted incidence for males was 1.96, 0.25, and 1.70 per 100,000 population, respectively. The age-adjusted incidence for females was 1.58, 0, and 1.58 per 100,000 population, respectively (Table 3). The median number of months from onset of symptoms to diagnosis of IBD was 11.04months (interquartile range, 1–120 months).

**Table 2:** Crude incidence rate (per 100,000) of IBD overall, CD, and UC in North of China.

	N	Crude incidence(per 100,000 persons)(95%CI)		
		Total	Male	Female
IBD	27	2.01(1.32–2.92)	2.12(1.16–3.56)	1.90(1.01–3.25)
UC	25	1.86(1.20–2.75)	1.82(0.94–3.18)	1.90(1.01–3.25)
CD	2	0.15(0.02–0.54)	0.30(0.04–1.09)	0.00(0.00–0.54)

**Table 3:** Age-standardized incidence rate (per 100,000) of IBD overall, CD, and UC in North of China.

	N	age-standardized incidence(per 100,000 persons)(95%CI)		
		Total	Male	Femal
IBD	27	1.77(1.16–2.59)	1.96(1.06–3.30)	1.58(0.84–2.70)
UC	25	1.64(1.06–2.43)	1.70(0.87–2.99)	1.58(0.82–2.70)
CD	2	0.13(0.02–0.47)	0.25(0.03–0.91)	0.00(0.00–0.54)

### 2. Clinical characteristics of new cases with IBD

The 2 CD patients experienced abdominal pain. Neither patient had diarrhea, blood or mucus in stool, but 1 patient had constipation. The median symptom duration before diagnosis was 61 months. All patients fall under L1 classification. Inflammation was found in both cases. One case was in remission and one case had moderate disease according to Harvey-Bradshaw activity index. One case had extra-intestinal manifestations of Arthralgia, but neither patient had other extra-intestinal manifestations like skin rashes, eye disease, ankylosing spondylitis, or primary sclerosing cholangitis (Table 4).

**Table 4:** Clinical characteristics of new cases of IBD in North of China.

	UC(n = 25)	CD(n = 2)
Symptom(%)		
Abdominal pain	13(52.0)	2(100.0)
Diarrhea	15(60.0)	0(0.0)
Constipation	3(12.0)	1(50.0)
Bloody stool	14(56.0)	0(0.0)
Mucus	15(60.0)	0(0.0)
Median time from symptom onset to diagnosis(range)	7.0(1–48)	61.0(2–120)
Extra-intestinal manifestations (%)		
Arthralgia	2(8.0)	1(50.0)
Skin rashes	1(4.0)	0(0.0)
Eye disease	1(4.0)	0(0.0)
Ankylosing spondylitis (%)	0(0.0)	0(0.0)
Promarysclerosing cholangitis(%)	0(0.0)	0(0.0)
Severity of UC(%)		
Mild	14(56.0)	1(50.0)
Moderate	11(44.0)	1(50.0)
Severe	0(0.0)	0(0.0)

Among 25 UC patients, 13 cases (52%) experienced abdominal pain, 15 cases (60%) experienced diarrhea, and 3 cases (12%) experienced constipation. Bloody stool was observed in 14 cases (56%), and mucus in stool was observed in 15 cases (60%). Average symptom duration before diagnosis was 7.04±12.6 months. Among 25 cases (24%) with UC, 6 cases had proctitis, 14 cases (56%) had left-sided colitis, and 5 cases (20%) had extensive or pancolitis. 14 cases (56%) had mild disease and 11(44%) had moderate disease according to the Mayo clinical index. 4 patients had extra-intestinal manifestations including 2 cases had arthralgia, 1 case had skin rashes, and 1 case had eye disease. No patients had ankylosing spondylitis or primary sclerosing cholangitis (Table 4).

## Discussion

In this study, we reported an annual incidence of IBD, CD, and UC for 1.77, 0.13, and 1.64 per 100,000 persons, respectively, in Northern China, which are obviously lower than that in western countries [2]–[5]. Moreover, the incidence of IBD, CD, and UC in Northern China is lower than that in Southern China [7]–[8]. For example, the incidence of IBD, CD, and UC in Guangzhou was 3.14, 1.09, and 2.05 per 100,000 persons, and 1.96, 0.51, and 1.45 per 100,000 persons in Wuhan. The reason of the lower IBD incidence in Daqing than in the other regions still need to be clarified, such as environmental factors, economic status and affordable medical service which will influence the case ascertainment. The variations on incidence of IBD from Northern China to Southern China may indicate a geographic difference. Guangzhou is located in Southern China on the Pearl River, having a humid subtropical climate, with a typical diet of seafood. Wuhan is located in Southern China at the intersection of the middle branches of the Yangtze and Han rivers, having humid subtropical climate with abundant rainfall. In contrast, Daqing is located in Northeastern China, known as the Oil Capital of China in the northern temperate zone, with a humid continental climate. Generally, winter is bitterly cold with occasional snowfalls, and spring and autumn are prevailed by monsoons. Therefore, there are many differences between Northern and Southern China including periods of sunshine, climate, diet, lifestyle, living conditions etc. However, this study provided no evidence to suggest which geographic features or environmental factors are responsible for the differences in incidence of IBD between Southern and Northern China. Although China has a large population of 1.3 billion, the incidence of IBD in China is obviously lower than that in western countries. IBD will place a heavy burden on medical costs, insurance, and quality of health care due to high relapse and remission rate of IBD patients.

Our study did not find the gender difference of UC occurrence, the ratio of male to female is 1.1∶1, which is difference from the other studies [11]–[13]. Also, it is hard to estimate the gender difference of CD occurrence because only 2 cases were found in one year. In western countries, UC was found more frequently in men (60%), whereas CD is 20%–30% more frequent in women [11]–[13]. Also, a similar male predominance of IBD was reported in the Southern Chinese population (male to female: 1.58 to 1 in UC and 2.4∶1 in CD) [7]. Due to low numbers of new cases in this study, it is difficult to analyze the detailed reasons for different gender trends in IBD in China and the real gender predominance under different physiological age groups.

In this study, the analysis of clinical characteristics of new IBD cases in the Northern Chinese population showed that diarrhea and abdominal pain were the most common symptoms in UC, which was similar to that of the Southern Chinese population. The main disease phenotype in UC patients is left-side colitis (56%) followed by proctitis (24%), which was also similar to that in the Southern Chinese population (41.9% for left-side colitis, 35.5% for proctitis in Guangzhou; 41% for left-side colitis, 35% for proctitisin Wuhan). 5 patients (18.5%) showed extra-intestinal manifestations of IBD in the Northern Chinese population, which is less common compared to the study in Guangzhou in Southern China (38%). In addition, the severity of IBD in the Northern Chinese population was not as severe as that in the Southern Chinese population, such that no patient with severe IBD was observed in the Northern population, but 6.5% severe activity was observed in UC in the Southern Chinese population. We also observed significant differences in the median diagnostic age (48 years in UC, 39 years in CD in Northern Chinese population *vs*. 40.6 years in UC, and 25.5 years in CD in Southern Chinese population and the duration from symptom onset to diagnosis (61 months in CD and 7 months in UC in Northern Chinese population *vs*. 25.6 months in CD and 17.3 months in Southern Chinese population) was different between Northern and Southern Chinese populations.

The major strengths of this study are the prospective population-based design, large sample size, and the completeness of IBD ascertainment and definition. However, this study still has some potential weaknesses. Although there is a well-established health care insurance system in Daqing, some patients do not take care of their health to see the doctor even though they have gastrointestinal symptoms, while some patients are more willing to acquire diagnosis and treatment from other cities with more skilled medical specialists. These problems may lead to possible selection bias and an underestimation in the proposed incidences. Understanding the most common clinical characteristics in different geographic areas may provide insights into possible etiologies of IBD. All of these observations highlight the need to compare environmental, dietary, and genotypic data in the future in different regions worldwide.
